# Improving rehabilitation motivation and motor learning ability of stroke patients using different reward strategies: study protocol for a single-center, randomized controlled trial

**DOI:** 10.3389/fneur.2024.1418247

**Published:** 2024-05-31

**Authors:** Jingwang Zhao, Jiangling Guo, Yeping Chen, Wenxi Li, Ping Zhou, Guangyue Zhu, Peipei Han, Dongsheng Xu

**Affiliations:** ^1^School of Rehabilitation Science, Shanghai University of Traditional Chinese Medicine, Shanghai, China; ^2^Graduate School, Shanghai University of Traditional Chinese Medicine, Shanghai, China; ^3^College of Rehabilitation Sciences, Shanghai University of Medicine and Health Sciences, Shanghai, China; ^4^The Second Rehabilitation Hospital of Shanghai, Shanghai University of Traditional Chinese Medicine, Shanghai, China; ^5^Yueyang Hospital of Integrated Traditional Chinese and Western Medicine, Shanghai University of Traditional Chinese Medicine, Shanghai, China; ^6^Zhongshan Hospital, Fudan University, Shanghai, China; ^7^Engineering Research Center of Traditional Chinese Medicine Intelligent Rehabilitation, Ministry of Education, Shanghai, China; ^8^Institute of Rehabilitation Medicine, Shanghai University of Traditional Chinese Medicine, Shanghai, China

**Keywords:** rehabilitation, reward, motivation, motor learning, stroke

## Abstract

**Background:**

Stroke survivors often face challenges in motor learning and motivation during rehabilitation, which can impede their recovery progress. Traditional rehabilitation methods vary in effectiveness, prompting the exploration of novel approaches such as reward strategies. Previous research indicates that rewards can enhance rehabilitation motivation and facilitate motor learning. However, most reward paradigms have utilized fixed reward amounts, which also have limitations. Exploring alternative, more effective reward strategies, such as probabilistic rewards, is warranted to optimize stroke patient rehabilitation.

**Methods:**

A total of 81 stroke patients will be recruited and randomly assigned to control, fixed reward, or probabilistic reward groups at a ratio of 1:1:1 using a randomized number table method. Participants will undergo 10 days of daily hand motor function rehabilitation training, with sessions lasting 20 min each. The training will involve pegboard tests and box and block tests. Control group participants will receive standard training, while fixed reward group members will receive monetary incentives for completing tests, and probabilistic reward group members will have the chance to win monetary rewards through a lottery box. Rehabilitation motivation and motor performance and functional near-infrared spectroscopy brain imaging will be conducted at designated time points. The primary outcome measure is the stroke rehabilitation motivation scale, and the second outcome measures include motor performance, simple test for evaluating hand function, motivation and pleasure scale self-report, and Pittsburgh rehabilitation participation scale.

**Discussion:**

Reward-based training enhance rehabilitation participation and adherence, it also improve motor learning speed and memory retention of stroke patients. The fixed reward applied in the past studies could diminish the sensitivity of stroke patients to rewards, while probabilistic reward may provide unpredictable or variable incentives or reinforcements for motor rehabilitation. This study will compare the efficacy of different reward strategies in enhancing motor learning ability and rehabilitation motivation among stroke patients. By conducting a randomized controlled trial, the study seeks to provide valuable insights into optimizing stroke rehabilitation protocols and improving patient outcomes.

**Clinical Trial Registration:**https://www.chictr.org.cn/, ChiCTR2400082419.

## Introduction

Stroke is a leading cause of long-term disability worldwide, often resulting in motor impairments that significantly impact the quality of life of affected individuals (1). Successful rehabilitation following stroke relies not only on the effectiveness of interventions but also on patients’ motivation to engage in therapy and their ability to learn and retain new motor skills (2). However, stroke survivors frequently experience challenges in motor learning due to neuroplastic changes in the brain and may lack motivation to participate in rehabilitation activities (3).

Motor learning is a complex process involving the acquisition, retention, and transfer of new motor skills through practice and experience. Stroke survivors often exhibit impairments in motor learning due to disruptions in neural circuits, alterations in sensorimotor integration, and impaired motor planning. Traditional rehabilitation approaches focus on repetitive practice and task-specific training to promote motor recovery. However, variability in individual responsiveness to rehabilitation interventions highlights the need for innovative strategies to enhance motor learning outcomes in stroke patients (4).

Motivation plays a critical role in driving engagement and adherence to rehabilitation programs. Stroke survivors may face various barriers to motivation, including frustration, lack of interest, and perceived lack of progress (5, 6). Providing meaningful incentives and rewards has been shown to enhance motivation and promote active participation in rehabilitation activities. Reward-based strategies, such as positive reinforcement, goal-setting, music and feedback, can help increase patients’ motivation and adherence to therapy regimens (7, 8).

Reward strategies have been increasingly integrated into rehabilitation programs to enhance patient motivation and engagement. Positive reinforcement, in the form of praise, encouragement, or tangible rewards, can reinforce desired behaviors and increase the likelihood of their repetition (9–11). Goal-setting allows patients to establish clear objectives and track their progress, providing a sense of achievement and motivation to continue rehabilitation efforts. Feedback mechanisms, such as visual or auditory cues, provide real-time information about performance, enabling patients to adjust their efforts and improve motor learning outcomes (12). However, in the majority of previous studies, the reward approach has been predominantly fixed. Fixed reward involves providing consistent and predictable incentives or reinforcements for desired behaviors or achievements. Examples of fixed rewards in stroke rehabilitation include tokens, or tangible rewards given consistently for meeting specific therapy goals or milestones. As time progresses, the sensitivity of participants to rewards gradually diminishes, which appears to limit the effectiveness of rewards. In contrast, another reward strategy, probabilistic reward, entails providing unpredictable or variable incentives or reinforcements for desired behaviors. This might include occasional praise, surprise rewards, or lottery-based incentives for demonstrating progress in therapy. Fixed rewards provide clear expectations and reinforcement for desired behaviors, promoting motivation and adherence to therapy regimens. On the other hand, probabilistic rewards create excitement and anticipation, stimulating motivation and engagement in therapy activities (13).

Previous studies have explored the effectiveness of fixed reward-based training in stroke rehabilitation, but there remains a need for comparative research to determine which approach—fixed or probabilistic reward—is more sensitive and efficient. The optimal types and delivery methods of rewards in stroke rehabilitation remain underexplored. Addressing this gap in the literature will provide valuable insights into optimizing stroke rehabilitation protocols and ultimately improving patient outcomes. This study aims to compare the efficacy of fixed and probabilistic reward strategies in enhancing motor learning ability and rehabilitation motivation in stroke patients, and seeks to inform the development of evidence-based rehabilitation protocols and improve patient outcomes following stroke.

## Methods and analysis

### Study design

This clinical trial adopts a single-center, single-blind randomized controlled design to investigate the efficacy of different reward strategies in enhancing motor rehabilitation outcomes among stroke patients. The study aims to enroll a total of 81 participants with residual motor deficits in their right upper limbs following a stroke. Participants will be randomly assigned to control group, fixed reward group or probabilistic reward group. Allocation will be conducted using a computer-generated randomization sequence to ensure an equal distribution of participants across the groups. Throughout the intervention period, all participants will receive standard rehabilitation training along with hand motor function training (20 min per day). The hand training was conducted using the pegboard test and the Box and Block Test (BBT) combined with different reward strategies. Rehabilitation motivation, motor performance evaluations and objective assessments will be conducted at designated time points.

### Participants

**Figure 1:**
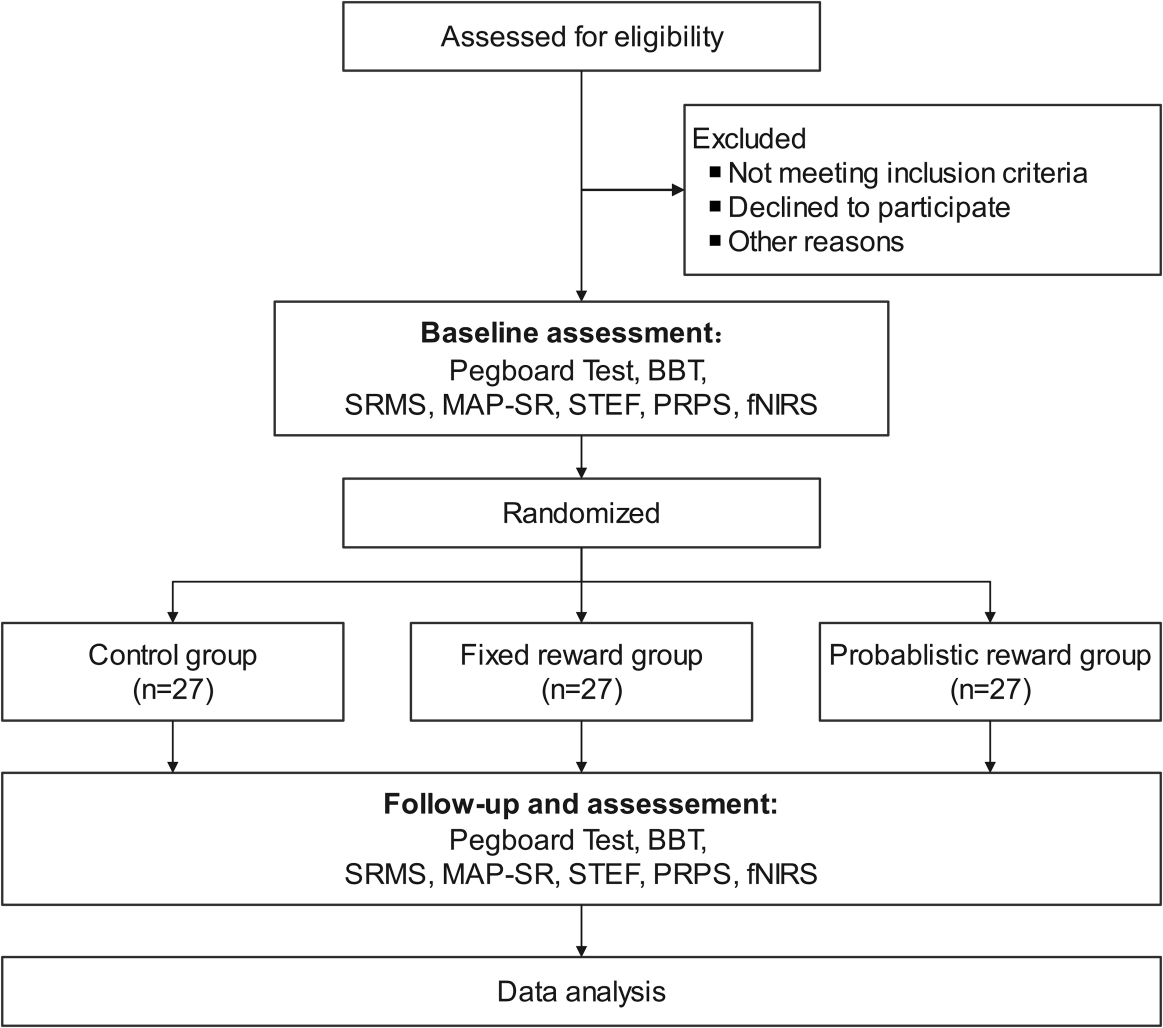
Flowchart of study design. BBT, Box and Block Test; fNIRS, Functional Near-Infrared Spectroscopy; MAP-SR, Motivation and Pleasure Scale Self-Report; PRPS, Pittsburgh Rehabilitation Participation Scale; SRMS, Stroke Rehabilitation Motivation Scale; STEF, Simple Test for Evaluating Hand Function.

This research will recruit subjects from the department of rehabilitation medicine at the Second Rehabilitation Hospital of Shanghai. Patient recruitment will commence on May 1, 2024. Recruitment methods will primarily include advertising, physician referrals, and internet recruitment. All subjects will be initially screened by the principal investigator before enrollment. Patients who meet the recruitment criteria and express interest will be individually informed of the trial details and consent requirements (Figure 1). Patients will sign a paper version of the informed consent form after fully understanding the benefits and risks of this study.

**Inclusion Criteria**: (1) Patients diagnosed with stroke based on clinical assessment and comprehensive imaging examinations. First onset of stroke; duration of illness ≥1 month. (2) The impairment of motor function in the right upper limb (including the forearm and hand). (3) Age between 18 and 80 years, irrespective of gender, and right-handed. (4) Brunnstrom stages III to V. (5) Able to tolerate assessment sessions lasting from half an hour to one hour. (6) Participants and their legally authorized guardians understand and agree to participate in this study, and jointly sign the informed consent form.

**Exclusion Criteria**: (1) Patients with severe systemic diseases such as cardiopulmonary disorders, uremia or heart failure that render them unable to tolerate rehabilitation treatment. (2) Diagnosed with psychiatric disorders, severe depression, or having a family history of psychiatric disorders (HAMD-17 scores > 24). (3) Severe joint contractures (active range of motion of the upper limb is less than half of the normal range without eliciting pain). (4) Presence of consciousness disorders due to any cause, Glasgow coma score < 15 (14). (5) Auditory or visual impairments that may affect assessment and treatment. (6) Use of medications altering cortical excitability (antiepileptic drugs, sedatives, etc.). (7) Significant pain or mental disorders (visual analog scale > 6).

### Sample size

The statistical software G-power (Heinrich Heine University Düsseldorf, Dusseldorf, Germany) was utilized to ascertain the sample size, with an alpha value set at 0.05, power at 0.95. By employing Cohen’s d using data extracted from a prior study by Chen et al. (15), we ascertain the mean discrepancy between groups to be 2.34 (27.54–25.2), with a pooled standard deviation approximating 3.47. Acknowledging the discrepancy in sample sizes between the two groups, the effect size is computed to be 0.674. With these parameters, the calculated sample size per group was determined to be 23 participants, summing up to 69 participants across three groups. Anticipating a dropout rate of 15%, the final sample size was adjusted, necessitating 27 participants per group, resulting in a total sample size of 81 participants.

### Randomization, allocation, and blinding

The randomization procedure employed in this study adhered to rigorous standards, utilizing a randomized number table method. Subjects were allocated randomly into three groups (control group, fixed reward group and probabilistic reward group) at a precise ratio of 1:1:1, ensuring that each group consisted of 27 participants. Group allocation was meticulously executed using the sealed envelope method. Prior to participant assignment, random allocation sequences were meticulously generated and securely housed within sequentially coded, sealed, opaque envelopes. Following confirmation of subject eligibility by the researcher, envelopes were systematically unsealed, and subjects were then allocated to their respective groups.

In adherence to stringent confidentiality protocols, prior to envelope unsealing, the names and comprehensive profiles of eligible subjects were meticulously transcribed onto the exterior of each envelope. Duplicate records were also securely enclosed within, serving as a safeguard against any potential data loss or discrepancies. Furthermore, to ensure the utmost confidentiality and integrity of the allocation process, each envelope was reinforced with an additional layer of protection, such as a hardboard or tin foil insert, effectively shielding its contents from visibility under intense lighting conditions.

To uphold the scientific rigor of the study and mitigate the risk of bias, a single-blinding approach was meticulously implemented. This method ensured that assessors remained blinded to the subjects’ group allocations and the specific intervention protocols administered. Moreover, to maintain consistency and objectivity in the evaluation process, all patient training sessions and subsequent assessments were meticulously recorded. These assessments were conducted by therapists who were intentionally kept unaware of the patients’ group assignments. In case of anomalous data detected during data analysis or data verification, the designated data analyst will promptly notify both the principal investigator and the assessor. Subsequently, a comprehensive review of the recorded sessions will be conducted to verify the accuracy and reliability of the obtained results, thereby upholding the highest standards of scientific integrity and validity.

### Intervention

Following the completion of the informed consent process, participants will formally commence the trial protocol. Throughout the inpatient period, hand motor function rehabilitation training will be administered once daily, with each session lasting approximately 20 min. This regimen comprises three Pegboard Tests and three BBT. In the context of the pegboard test, a common assessment tool used in rehabilitation settings, motor performance is evaluated by measuring the time it takes for individuals to complete specific tasks involving moving pegs from one location to another on a pegboard. The pegboard test often varies in complexity, with tasks ranging from simple movements to more challenging ones requiring precise coordination and dexterity. The Pegboard Test will be stratified into three difficulty tiers based on training complexity: peg removal, peg insertion, and peg flipping, further classified into three levels according to peg size.

The training regimen encompasses two distinct phases. In phase one, known as the baseline assessment, the Pegboard Test evaluates the maximum difficulty level of actions that patients can successfully complete and identifies the minimum peg size. BBT training entails patients transferring blocks as swiftly as possible within a 1-min timeframe, with a 1-min inter-session rest period, during which the number of successfully transferred blocks is recorded.

Phase two entails rehabilitation training, wherein patients undergo hand motor training once daily for 10 consecutive days, employing the highest difficulty level of actions and pegs. Patients are tasked with completing all pegs expeditiously. Following each training session, there will be a 1-min rest period, after which the pegboard will be reset for three additional pegboard training sessions. Patient metrics, including maximum action difficulty, peg size, and completion time, will be meticulously documented. BBT training will adhere to the same protocol as the baseline assessment, recording the number of successfully transferred blocks within a 1-min duration, repeated thrice.

Participants assigned to the control group will complete the training regimen as stipulated, devoid of any reward system. Conversely, participants in the fixed reward group will receive a 5 RMB incentive for each completed test prior to pegboard and box block trials, culminating in a total reward of 30 RMB upon completion of three pegboard tests and three BBT sessions daily. Those allocated to the probabilistic reward group will obtain a lottery opportunity following each completed experiment, entailing monetary rewards ranging from 1, 2, 5, 10, and 15 RMB, with an average probability reward of approximately 5 RMB. Consequently, completing three pegboard tests and three BBT sessions daily may yield rewards ranging from 6 RMB to 90 RMB. We prepared a square paper box to serve as a dark box for drawing lots. There is a total of 70 ping-pong balls inside, with 20 balls labeled with the number 1, 16 balls labeled with the number 2, 16 balls labeled with the number 5, 10 balls labeled with the number 10, and 8 balls labeled with the number 15. The amount of money awarded corresponds to the number marked on the ping-pong ball.

To ensure consistency and quality of the intervention measures throughout the trial, a detailed intervention protocol will be developed outlining the procedures, techniques, and timing for implementing different reward strategies. This protocol will serve as a guide for all interventionists to ensure uniformity in the delivery of interventions. Interventionists will be required to accurately document the delivery of each intervention session using standardized forms. This documentation will include details such as the content of the intervention, duration, participant responses, and any deviations from the protocol. The research team aims at ensure consistency and quality in the delivery of intervention measures. Maintaining intervention fidelity is crucial for accurately evaluating the effectiveness of different reward strategies in improving motor learning ability and rehabilitation motivation among stroke patients.

The safety and well-being of participants will be closely monitored throughout the duration of the study. Adverse events and serious adverse events will be promptly reported to the relevant regulatory authorities and the ethics committee, and appropriate measures will be taken to ensure participant safety.

### Outcome assessment

The components of the assessment include basic information (gender, age, duration of illness, medication history, etc.), motivation, motor performance and objective assessments. The main assessments focus on motivation and motor performance. Comprehensive video recordings of each training session will be subjected to meticulous analysis to evaluate daily motor performance. Rehabilitation motivation evaluations and functional near-infrared spectroscopy (fNIRS) measurements will be conducted at designated time points (Table 1).

**Table 1:** Recommended content for the schedule of enrolment, interventions, and assessments.

	Study period						
	Enrolment and assessment	Allocation	Training			Follow-up	
Timepoint	−D_1_	0	D_1_	D_2_–D_9_	D_10_	D_14_	D_28_
Enrolment							
Eligibility screen	X						
Informed consent	X						
Allocation		X					
Interventions							
Control group			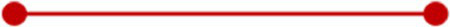				
Fixed reward group			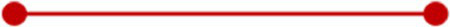				
Probabilistic reward group			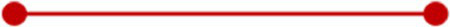				
Assessments							
SRMS	X		X		X	X	X
MAP-SR	X		X		X	X	X
PRPS	X		X		X	X	X
Motor performance	X		X	X	X	X	X
fNIRS	X		X		X	X	X

### Primary outcomes

The Stroke Rehabilitation Motivation Scale (SRMS) is a validated assessment tool developed by Australian scholar White in 2012, specifically tailored to measure the motivation levels of stroke patients engaging in rehabilitation programs (16). It offers a structured approach to evaluate various dimensions of motivation essential for effective rehabilitation. The scale comprises seven dimensions, including amotivation, external regulation, introjected regulation, identified regulation, integrated regulation, intrinsic motivation to know, and intrinsic motivation to accomplish. Each dimension represents different aspects of motivation, ranging from external factors like rewards or obligations to internal factors such as inherent satisfaction and personal goals. It consists of 28 items, including both positively and negatively phrased statements, allowing for a nuanced assessment of motivation levels. The SRMS serves as a valuable tool in rehabilitation practice, enabling clinicians to assess, monitor, and address motivational barriers to maximize the effectiveness of stroke rehabilitation programs.

### Secondary outcomes

The second outcomes include motor performance, STEF, MAP-SR, and PRPS.

Motor performance assessment involves evaluating the ability of individuals to execute motor tasks effectively. This assessment typically encompasses factors such as the level of task difficulty, characteristics of the equipment used (e.g., peg size), and the time taken to complete tasks. The Simple Test for Evaluating Hand Function (STEF) is a valuable assessment tool specifically designed to evaluate hand and upper limb function. Developed initially by Kaneko Tsubasa in Japan and subsequently adapted for use in the United States (17). The STEF provides objective measurements of performance, allowing clinicians to track progress over time and compare results to normative data. The Motivation and Pleasure Scale Self-Report (MAP-SR) is an assessment tool originally developed by Llerena et al. (18). The MAP-SR encompasses items that probe different dimensions of motivation and pleasure, allowing for a comprehensive understanding of an individual’s subjective experiences. The Pittsburgh Rehabilitation Participation Scale (PRPS) is a vital assessment tool developed by Professor Lenze EJ at the University of Pittsburgh in 2004 (19). The scale offers a structured and systematic approach to assess the extent to which individuals engage in and contribute to their rehabilitation process during inpatient treatment. The PRPS serves as an indispensable tool for rehabilitation specialists, enabling us to assess, promote, and monitor patient participation in rehabilitation programs.

Functional Near-Infrared Spectroscopy (fNIRS) serves as a valuable tool for assessing brain activity and hemodynamic responses during motor tasks in stroke patients. During the assessment, participants will engage in motor tasks, such as hand function exercises, while fNIRS sensors are placed over predetermined cortical areas. Researchers can quantitatively evaluate the effectiveness of different reward strategies in modulating cortical activation patterns, thus elucidating their impact on motor learning ability and rehabilitation motivation in stroke patients.

Regular quality control checks will be conducted throughout the trial to monitor the consistency and accuracy of assessments. This will involve periodic reviews of assessment videos or live observations by a designated quality control team to identify any discrepancies or issues in assessment administration. Feedback will be provided to assessors, and additional training or clarification will be offered if necessary to rectify any identified issues.

### Data collection and data management

Ensuring the precision and trustworthiness of research data stands as a cornerstone in clinical studies. A methodical process encompassing data collection, verification, locking, and unblinding is meticulously employed to uphold rigorous data quality standards. At the outset, paper-based Case Report Forms (CRFs) and specialized assessment scales are meticulously employed to mitigate potential human errors during data collection. Each CRF undergoes meticulous transcription into the database by two independent personnel, with any disparities promptly rectified using software tools to augment efficiency and establish a resilient data foundation. During the data verification phase, spearheaded by a dedicated data administrator, stringent protocols are enacted to meticulously identify and rectify any errors or inconsistencies in the data entry process. This phase is of paramount importance in ensuring the precision and reliability of the dataset. Subsequent to verification, the data is securely locked to safeguard its integrity and prevent unauthorized alterations without unanimous agreement. This procedure serves to uphold the sanctity and fidelity of the dataset throughout the analysis phase. In the pivotal unblinding process, statistical analysis unveils the treatment modalities received by participants, subsequently followed by the disclosure of treatment group statuses. Transparency and impartiality in this phase are of utmost importance in upholding the scientific integrity and credibility of the trial results.

Overall, this rigorous data collection and management protocol are devised to furnish invaluable insights into the efficacy of diverse reward strategies in stroke rehabilitation. By adhering to exacting data quality standards, the study aspires to contribute significantly to the refinement of rehabilitation protocols tailored for stroke patients.

### Statistical analysis

Categorical variables like gender and affected side will be presented as percentages and compared using the chi-square test. Continuous variables will undergo normality assessment with the Shapiro–Wilk test and variance homogeneity examination with Levene’s test. One-Way ANOVA will compare motivation among groups before and after training, followed by Tukey’s HSD test for *post hoc* comparisons. The repeated measures ANOVA was performed to analyze changes in rehabilitation motivation scales data and average time spent in pegboard test and BBT in the three groups. Non-normal or heterogeneous data will be analyzed using the Kruskal-Wallis test, with *post hoc* tests conducted using Dunn’s test. Normal data will be presented as mean ± standard deviation, while non-normal data will be presented as median and quartiles. The significance level (P) for hypothesis testing will be set at 0.05.

## Discussion

Stroke-induced motor impairments pose significant challenges to patients’ quality of life and independence, necessitating effective rehabilitation strategies. As highlighted in the introduction, stroke survivors often experience difficulties in motor learning due to neuroplastic changes in the brain, coupled with motivational barriers that hinder active participation in rehabilitation activities (20, 21). The incorporation of reward strategies, such as positive reinforcement and goal-setting, aims to address these challenges by enhancing motivation and engagement in therapy sessions (22).

Previous studies have consistently demonstrated the efficacy of motivational interviews and reward-based training in bolstering the rehabilitation motivation of individuals with neurological injuries. For instance, findings from various intervention studies revealed a significant positive effect of motivational interviewing on patient engagement and adherence to rehabilitation programs (23–25). Similarly, comparing traditional rehabilitation approaches with reward-based training found that participants in the reward group exhibited higher levels of motivation and persistence in completing motor tasks (26). Furthermore, Studies have elucidated the neural mechanisms underlying the influence of rewards on motivation, highlighting the activation of dopaminergic pathways and the striatum during reward anticipation, which in turn enhances motivation and learning processes (27). Collectively, these findings underscore the importance of incorporating motivational strategies and reward systems into neurorehabilitation protocols to optimize patient outcomes and facilitate recovery.

Earlier studies demonstrated that reward alone did not enhance the learning rate in the VMR task, whereas reward combined with punishment not only accelerated the learning rate but also increased the learning extent (28, 29). Nikooyan et al. suggested that reward feedback alone can drive motor adaptation (without increasing the rate beyond that of the control group), and the combination of reward and sensory feedback accelerates learning (30). It was also supported that reward alone boosts or accelerates learning speed in sequence learning paradigms. The participants with monetary incentives have a higher learning rate in a discrete motor sequence task because the reward enhances motivation (31). Additionally, Sebastian Sporn et al. dissociated the effects of different types of rewards, namely, performance feedback and monetary incentives, through a novel motor task, and the results demonstrated that monetary incentives alone rapidly shortened movement time, whereas feedback after correct responses primarily improved learning-related movement time performance. Importantly, pairing both monetary incentives and feedback after correct responses enhanced movement time performance and improved fusion of movements (12). The majority of studies investigating the impact of rewards on motor learning mentioned above were conducted in healthy participants, employing fixed reward paradigms. It wasn’t until 2017 that, in a visuomotor rotation-based reaching task utilizing a robotic arm, the reward group of chronic stroke patients exhibited superior adaptation and readaptation compared to the neutral group, and finally, the reward group showed greater retention. Quattrocchi et al. were the first to provide evidence that reward and punishment can augment motor adaptation in stroke patients (32). This finding was corroborated by a recent study which was also conducted in stroke patients (33). However, the rewards utilized in these investigations remained fixed. To date, there is still a lack of randomized controlled trials examining the effects of fixed versus probabilistic rewards on motor rehabilitation in stroke patients. The study we are undertaking aims to address this gap.

The comparison between fixed and probabilistic reward strategies provides valuable insights into their respective efficacy in promoting motor learning and rehabilitation motivation among stroke patients. This study will contribute to the growing body of evidence on the effectiveness of reward-based interventions in stroke rehabilitation. By elucidating the differential impacts of fixed and probabilistic rewards on patient outcomes, this research informs the development of tailored rehabilitation protocols that optimize motivation and motor learning outcomes in stroke survivors. The novelty of this study lies in its comparative approach, which directly assesses the relative effectiveness of fixed versus probabilistic reward strategies in stroke rehabilitation. In addition to its clinical implications, this study contributes to theoretical models of motivation and learning in rehabilitation settings. By elucidating the mechanisms underlying the effectiveness of reward-based strategies, such as reinforcement schedules and expectancy theory, this research may enhance our understanding of the psychological factors that influence patient engagement and adherence to rehabilitation programs.

Overall, this study underscore the importance of integrating motivational strategies into stroke rehabilitation protocols to enhance motor learning ability and rehabilitation motivation. By identifying the most effective reward strategies for stroke patients, this research has the potential to inform clinical practice and improve the effectiveness of rehabilitation interventions.

## Ethics statement

The studies involving humans were approved by Ethics Committee of Shanghai Second Rehabilitation Hospital of Shanghai. The studies were conducted in accordance with the local legislation and institutional requirements. The participants provided their written informed consent to participate in this study.

## Author contributions

JZ: Visualization, Formal analysis, Writing – review & editing, Writing – original draft, Methodology, Investigation, Conceptualization. JG: Writing – original draft, Software, Methodology, Investigation. YC: Writing – review & editing, Supervision, Investigation, Data curation. WL: Writing – review & editing, Software, Methodology, Conceptualization. PZ: Writing – review & editing, Methodology, Conceptualization. GZ: Writing – review & editing, Methodology. PH: Writing – review & editing, Supervision, Conceptualization. DX: Writing – review & editing, Funding acquisition, Validation, Supervision, Project administration, Conceptualization.

## References

[1] Langhorne P Bernhardt J Kwakkel G. Stroke rehabilitation. Lancet. (2011) 377:1693–702. doi: 10.1016/S0140-6736(11)60325-5 21571152

[2] Winstein CJ Stein J Arena R Bates B Cherney LR Cramer SC . Guidelines for adult stroke rehabilitation and recovery: a guideline for healthcare professionals from the American Heart Association/American Stroke Association. Stroke. (2016) 47:e98–e169. doi: 10.1161/STR.0000000000000098, PMID: 27145936

[3] Chen Y Abel KT Janecek JT Chen Y Zheng K Cramer SC. Home-based technologies for stroke rehabilitation: a systematic review. Int J Med Inform. (2019) 123:11–22. doi: 10.1016/j.ijmedinf.2018.12.001, PMID: 30654899 PMC6814146

[4] Stinear CM Lang CE Zeiler S Byblow WD. Advances and challenges in stroke rehabilitation. Lancet Neurol. (2020) 19:348–60. doi: 10.1016/S1474-4422(19)30415-6 32004440

[5] Salter KL Foley NC Jutai JW Teasell RW. Assessment of participation outcomes in randomized controlled trials of stroke rehabilitation interventions. Int J Rehabil Res. (2007) 30:339–42. doi: 10.1097/MRR.0b013e3282f144b7 17975455

[6] Wagner F Rogenz J Opitz L Maas J Schmidt A Brodoehl S . Reward network dysfunction is associated with cognitive impairment after stroke. Neuroimage Clin. (2023) 39:103446. doi: 10.1016/j.nicl.2023.103446, PMID: 37307650 PMC10276182

[7] Sihvonen AJ Sarkamo T Leo V Tervaniemi M Altenmuller E Soinila S. Music-based interventions in neurological rehabilitation. Lancet Neurol. (2017) 16:648–60. doi: 10.1016/S1474-4422(17)30168-0 28663005

[8] Doppler C Meyer L Dovern A Stuhmer-Beckh J Weiss PH Fink GR. Differential impact of social and monetary reward on procedural learning and consolidation in aging and its structural correlates. Front Aging Neurosci. (2019) 11:188. doi: 10.3389/fnagi.2019.00188, PMID: 31417395 PMC6682642

[9] Sadnicka A Edwards MJ. The influence of reward and punishment on motor learning. Mov Disord. (2015) 30:1724. doi: 10.1002/mds.26372, PMID: 26256449

[10] Lutz K Pedroni A Nadig K Luechinger R Jancke L. The rewarding value of good motor performance in the context of monetary incentives. Neuropsychologia. (2012) 50:1739–47. doi: 10.1016/j.neuropsychologia.2012.03.030, PMID: 22569215

[11] Summerside EM Shadmehr R Ahmed AA. Vigor of reaching movements: reward discounts the cost of effort. J Neurophysiol. (2018) 119:2347–57. doi: 10.1152/jn.00872.2017, PMID: 29537911 PMC6734091

[12] Sporn S Chen X Galea JM. The dissociable effects of reward on sequential motor behavior. J Neurophysiol. (2022) 128:86–104. doi: 10.1152/jn.00467.2021, PMID: 35642849 PMC9291426

[13] Kapogiannis D Campion P Grafman J Wassermann EM. Reward-related activity in the human motor cortex. Eur J Neurosci. (2008) 27:1836–42. doi: 10.1111/j.1460-9568.2008.06147.x 18371077 PMC5456265

[14] Teasdale G Jennett B. Assessment of coma and impaired consciousness. A practical scale. Lancet. (1974) 304:81–4. doi: 10.1016/s0140-6736(74)91639-0 4136544

[15] Chen HM Lee HL Yang FC Chiu YW Chao SY. Effectiveness of motivational interviewing in regard to activities of daily living and motivation for rehabilitation among stroke patients. Int J Environ Res Public Health. (2020) 17:17. doi: 10.3390/ijerph17082755, PMID: 32316197 PMC7216097

[16] White GN Cordato DJ O'Rourke F Mendis RL Chan DKY. Validation of the stroke rehabilitation motivation scale: a pilot study. Asian J Gerontol Geriatr. (2012) 7:80–7. https://www.researchgate.net/publication/286804560_Validation_of_the_Stroke_Rehabilitation_Motivation_Scale_A_pilot_study

[17] Kaneko T Muraki T. Development and standardization of the hand function test In: Bulletin of allied medical sciences Kobe Bams (1990)

[18] Llerena K Park SG McCarthy JM Couture SM Bennett ME Blanchard JJ. The motivation and pleasure scale-self-report (map-sr): reliability and validity of a self-report measure of negative symptoms. Compr Psychiatry. (2013) 54:568–74. doi: 10.1016/j.comppsych.2012.12.001, PMID: 23351831 PMC4762003

[19] Lenze EJ Munin MC Quear T Dew MA Rogers JC Begley AE . The Pittsburgh rehabilitation participation scale: reliability and validity of a clinician-rated measure of participation in acute rehabilitation. Arch Phys Med Rehabil. (2004) 85:380–4. doi: 10.1016/j.apmr.2003.06.001, PMID: 15031821

[20] Olgiati E Russell C Soto D Malhotra P. Motivation and attention following hemispheric stroke. Prog Brain Res. (2016) 229:343–66. doi: 10.1016/bs.pbr.2016.06.011, PMID: 27926447

[21] Rochat L Van der Linden M Renaud O Epiney JB Michel P Sztajzel R . Poor reward sensitivity and apathy after stroke: implication of basal ganglia. Neurology. (2013) 81:1674–80. doi: 10.1212/01.wnl.0000435290.49598.1d, PMID: 24107869

[22] Gangwani R Cain A Collins A Cassidy JM. Leveraging factors of self-efficacy and motivation to optimize stroke recovery. Front Neurol. (2022) 13:823202. doi: 10.3389/fneur.2022.823202, PMID: 35280288 PMC8907401

[23] Lal S Korner-Bitensky N. Motivational interviewing: a novel intervention for translating rehabilitation research into practice. Disabil Rehabil. (2013) 35:919–23. doi: 10.3109/09638288.2012.711897, PMID: 22991934

[24] Oyake K Suzuki M Otaka Y Momose K Tanaka S. Motivational strategies for stroke rehabilitation: a delphi study. Arch Phys Med Rehabil. (2020) 101:1929–36. doi: 10.1016/j.apmr.2020.06.007 32753111

[25] Watkins CL Wathan JV Leathley MJ Auton MF Deans CF Dickinson HA . The 12-month effects of early motivational interviewing after acute stroke: a randomized controlled trial. Stroke. (2011) 42:1956–61. doi: 10.1161/STROKEAHA.110.602227, PMID: 21700946

[26] Vassiliadis P Derosiere G Dubuc C Lete A Crevecoeur F Hummel FC . Reward boosts reinforcement-based motor learning. Iscience. (2021) 24:102821. doi: 10.1016/j.isci.2021.102821, PMID: 34345810 PMC8319366

[27] Geisler CE Hayes MR. Metabolic hormone action in the vta: reward-directed behavior and mechanistic insights. Physiol Behav. (2023) 268:114236. doi: 10.1016/j.physbeh.2023.114236 37178855 PMC10330780

[28] Song Y Lu S Smiley-Oyen AL. Differential motor learning via reward and punishment. Q J Exp Psychol (Hove). (2020) 73:249–59. doi: 10.1177/1747021819871173, PMID: 31382855

[29] Johannsen WJ . Effect of reward and punishment on motor learning by chronic schizophrenics and normals. J Clin Psychol. (1962) 18:204–7. doi: 10.1002/1097-4679(196204)18:2<204::aid-jclp2270180229>3.0.co;2-s, PMID: 14451938

[30] Nikooyan AA Ahmed AA. Reward feedback accelerates motor learning. J Neurophysiol. (2015) 113:633–46. doi: 10.1152/jn.00032.2014, PMID: 25355957

[31] Anderson SP Adkins TJ Gary BS Lee TG. Rewards interact with explicit knowledge to enhance skilled motor performance. J Neurophysiol. (2020) 123:2476–90. doi: 10.1152/jn.00575.2019 32432504

[32] Quattrocchi G Greenwood R Rothwell JC Galea JM Bestmann S. Reward and punishment enhance motor adaptation in stroke. J Neurol Neurosurg Psychiatry. (2017) 88:730–6. doi: 10.1136/jnnp-2016-314728, PMID: 28377451

[33] Widmer M Held J Wittmann F Valladares B Lambercy O Sturzenegger C . Reward during arm training improves impairment and activity after stroke: a randomized controlled trial. Neurorehabil Neural Repair. (2022) 36:140–50. doi: 10.1177/15459683211062898, PMID: 34937456 PMC8796156

